# Go to Work Friends

**Published:** 1871-10

**Authors:** Charles S. Lewis


### GO TO WORK FRIENDS!

We take great pleasure in presenting to the readers of THE COMPANION the following letter, written by one of our Associate Editors—one of the ablest and purest, even of the Old Dominion. We especially invite the attention of our Associate and Corresponding Editors to the suggestions therein contained. We hope they will exert every effort to give *practical* effect to them. Will not every friend of THE COMPANION go to work for it? Will not every subscriber at least, obtain *one* additional name? Friends go to work!

DEAR COMPANION:

Allow me to come around the "editorial table" with my brother editors for a short "talk." I wish to open up a conversation with my brother "Associates" for the purpose of surveying the ground before us, that we may unitedly make THE COMPANION the true representative organ of the Profession of the South. THE COMPANION, for the object it has in view, (*i. e.*,) to be a compendium of the busy practitioner, is a success. It is the best periodical for the purpose I have yet seen. With the united energies of its friends it can be made to cover a broader field than that contemplated by its originators. While being devoted to culling the best current medical literature, it can also open up to our physicians the best medium through which they may reach each other. It is especially desirable to make it the organ of the Profession from the high ethical position it has taken, for the meritorious purpose of restoring the Profession to its former exalted rank among the learned professions of this country.

Such a Journal, advocating high principles, is imperatively demanded by the times. Do we not all feel and see this? Let us therefore, eagerly embrace the opportunity offered the Profession, by which a grand effort—a rallying of the true men—may be made for this great and noble purpose. In order to accomplish this, sound ethics must be proclaimed from the house-tops—they must be made to permeate all the high ways and by-ways of the Profession. We have the medium,—and we have an army of good and true men who are delighted in holding up the "golden rule" embodied in the written and unwritten ethical law, which must govern the Profession—what therefore, do we most need? I give it as my opinion that it is to make THE COMPANION *the Companion* indeed of every physician in the country. This can be done by a little work, unitedly by its friends—especially the Associate and Corresponding Editors. I am informed that should each of the editors secure *twenty* "bona fide" subscribers it will be financially a success. I have no doubt it will be sustained. A superior journal for the active practitioner I never yet saw, and it will be appreciated wherever it goes. Let it go where it will, the pnblisher deservers the thanks of the Profession. We do thank him for giving us such a medium, on his own responsibility—relying alone on the Profession to sustain him. Shall we disappoint him? I *say no!* but let us go to work and send him twenty subscribers within the next few weeks. It certainly would not do the Profession any injury. It would not lower its dignity for THE COMPANION to be read by the intelligent portion of every Profession. If the people could be made to believe the high moral and ethical principles which are to be found in every number of THE COMPANION, the doctorate would soon be again clothed in its former honors. If we really wish to do much good, a correct public sentiment must be formed, which can be done only by instructing the masses. Therefore, I will make a proposition to my brother Associate and Corresponding Editors—which is this: That each one agree to canvass, by letter and personal appeal, his own and every adjoining county of his immediate section—to interest as many personal friends to unite in this work, as soon as possible, to work for THE COMPANION as the common property of the Profession—to hold up the hands of our generous publisher, and make him, by postal orders, etc., feel that we are earnestly at work—that we will sustain him in the noble effort to supply such a journal as shall be a credit to himself and an honor to the Profession. In order to do this effectually, I propose to commence at once my efforts in its behalf. I would advise that every subscriber get one or more new ones. I admire the pluck and spirit of Dr. G. D. Hoge, of Freer, Ark. He says: I am willing to become one of any number of subscribers, that will be responsible for, at least, five additional names. Are there not a thousand in the Southern States, who are willing to do as much to sustain an independent, first-class, Medical Journal, as a common heritage to the Profession? If each editor will select 20 good and true reading men and request the publisher to send them a specimen number of THE COMPANION, we believe it will be done. Let us make the effort.

Yours, very cordially, CHARLES S. LEWIS.

